# In Situ Performic Acid Epoxidation of Polyfarnesene: Evidence of Oxirane Ring Instability and Its Impact on Multifunctional Polymer Composition

**DOI:** 10.3390/polym18070844

**Published:** 2026-03-30

**Authors:** Geilza A. Porto, Luiz Guilherme A. de Paula, Luciano N. Batista, Marcos L. Dias

**Affiliations:** 1 Instituto de Macromoléculas Professora Eloisa Mano (IMA), Universidade Federal do Rio de Janeiro, Av. Horacio Macedo, Bloco J, 2030, Rio de Janeiro 21941-598, RJ, Brazil; geilzaporto@ima.ufrj.br (G.A.P.); luizguilhermeacm@ima.ufrj.br (L.G.A.d.P.); 2 Instituto Nacional de Metrologia, Qualidade e Tecnologia (Inmetro), Avenida Nossa Senhora das Graças, 50, Duque de Caxias 25250-020, RJ, Brazil; lnbatista@inmetro.gov.br

**Keywords:** polyfarnesene, epoxidation, oxirane instability

## Abstract

Polyfarnesene, a bio-based polymer, was epoxidized in situ using performic acid to investigate oxirane ring formation, stability, and the role of its bottlebrush architecture in the kinetics. The reaction reached a maximum epoxidation degree of ~20% after 6 h but underwent side reactions, producing hydroxyl and formic ester groups. FTIR and ^1^H NMR revealed that ring opening began within the first hour, whereas residual unsaturated bonds persisted after prolonged reaction, owing to steric shielding by the polymer’s long C11–C13 side chains. Unlike smaller polydiene homologues, polyfarnesene exhibited slower ring-opening kinetics, retaining approximately 10% of oxirane groups after 20 h. GPC showed minimal molecular weight changes but an increase in polydispersity, confirming structural rearrangements without chain scission or crosslinking. DSC demonstrated that oxirane incorporation increased the T_g_; however, side reactions reduced this effect by limiting chain mobility. These findings establish that the spatial constraints imposed by the bottlebrush architecture of polyfarnesene govern the reaction kinetics, restricting epoxidation efficiency and favoring esterification pathways. This interplay provides a basis for designing bio-based polymers with tunable thermal properties. Controlling the reaction environment to suppress side reactions is key to producing high-T_g_ epoxidized derivatives suitable for rubber technologies and sustainable materials.

## 1. Introduction

In recent decades, there has been an increase in research on sustainable polymers and the development of bio-based rubbers to replace petroleum-derived resources [1,2,3]. Research on biomass-derived materials holds great potential for both materials science and industry. The alarming accumulation of plastic waste in the environment has led to growing environmental awareness and demand for sustainable alternatives based on raw materials from renewable sources, thereby reducing the use of fossil resources for their production [4].

One of the most promising materials is farnesene (7,11-dimethyl-3-methylene-1,6,10-dodecatriene), a bio-based substance derived from yeast fermentation of sugarcane raw materials [5,6]. Farnesene has been used as a raw material for the production of oils, cosmetics [7,8], surfactants, lubricants, fuels, and polymers [9]. Its high boiling point (260 °C) makes it particularly suitable for use as a green bio-based solvent, which is ideal for sustainable processes [10,11,12].

Polyunsaturated farnesenes can be converted into polyfarnesenes [13] using different polymerization methods, such as anionic, coordination, and free radical polymerization, and the polymer has even been vulcanized with common commercial additives to produce bio-based rubbers [14,15]. Polyfarnesene belongs to a homologous series of polydienes that includes polybutadiene (PB, no side chains), polyisoprene (PI, methyl side chains), polymyrcene (PM, C6–C8 side chains), and polyfarnesene (PF, C11–C13 side chains) [12,16,17]. Owing to their long side chains and highly branched “bottlebrush” structure, polyfarnesenes have the potential to improve the viscoelastic and rheological properties of composites [5,18]. Furthermore, their bulky architecture is expected to impose significant steric constraints on chemical modification reactions.

The presence of unsaturated bonds in the functionalized alkenyl side chains of polyfarnesene allows the creation of materials with superior thermal properties and relative chemical stability [1]. The double bonds can facilitate the oxidation process, although the presence of unsaturation allows multifunctionalization, opening new routes for new materials [14].

The functionalization of polyterpenes, such as polyfarnesene and polymyrcene, constitutes a fundamental strategy to further broaden the applicability of these materials. This can be achieved through the introduction of functional end-groups, copolymerization, or post-polymerization modification reactions [14,19]. The literature widely reports the chemical modification of analogous polymers derived from isoprene; for example, liquid polyisoprenes functionalized with reactive groups (such as anhydride and carboxyl) have been successfully applied to enhance the toughness, mechanical strength, and glass transition temperature (T_g_) of thermosetting polymer networks [20]. However, despite this robust literature validation, regarding the modification of isoprenic polymers, the study of polyterpenes with complex side chains still presents specific gaps.

Many studies have been dedicated to the functionalization of polyfarnesene and similar polymers, such as polymyrcene and liquid polyisoprenes, using several methods [16,21,22]. Thiol–ene addition and epoxidation have been demonstrated to impart thermal and chemical improvements [5,14,23]. In particular, epoxidation converts double bonds to oxirane rings, which are versatile organic functions capable of reacting with several compounds, allowing the formation of diols, esters, and ethers, among others [24]. Although epoxidation can be conducted through different pathways, such as the use of in situ dioxiranes, microemulsions, or enzymatic catalysis [25,26], classical methodologies involving peracids have not been extensively investigated for sesquiterpenes, as these reactions are strongly limited by macromolecular steric hindrance [16,23].

The epoxidation of natural dienes has been reported in some studies. Matic et al. [16] epoxidized 1,4-polymyrcenes, which are polymers with structures similar to polyfarnesene. Adjustable degrees of epoxidation were attained using meta-chloroperbenzoic acid as the epoxidation agent and dichloromethane as the solvent at 0 °C. The yield reached 98% of epoxidation, but the trisubstituted epoxide groups, which were remarkably stable against nucleophiles under basic conditions, crosslinked or hydrolyzed in the presence of acids. The problem with Matic’s methodology is the use of a quantitative halogenated reagent and the remarkable use of dichloromethane as a solvent. Anastasiou [27] investigated the epoxidation of polymyrcene using a classical procedure with acetic acid, hydrogen peroxide, and inorganic acid as the catalyst. Curiously, the yield of epoxidation was less than 10% after 4 h. It is important to mention that this reaction system has elevated yields when applied to the epoxidation of unsaturated vegetable oils [11,28]. This sharp contrast in reactivity between polymyrcene and vegetable oils, despite both containing unsaturated groups, suggests that polymer architecture plays a critical role in determining reaction outcomes, an aspect that remains poorly understood for higher homologues, such as polyfarnesene.

Although polyfarnesene has a structure similar to that of polymyrcene, it has a higher molar mass, and its compact bottlebrush architecture reduces the diffusion of reactants. Furthermore, while oxirane ring opening and side reactions are well-documented phenomena in peracid epoxidations [11,29,30,31,32,33], the extent to which the bottlebrush architecture of polyfarnesene influences the kinetics of oxirane degradation and the relative stability of the formed epoxy groups has not been systematically investigated. As reported by several authors, oxirane rings are susceptible to acidic and basic conditions, which can promote ring opening [30,34]. Consequently, within the broad scope of terpene polymer modification, no prior studies have reported the specific epoxidation of polyfarnesene using performic acid as an oxidizing agent; therefore, it is imperative to investigate how its peculiar brush-like structure responds to this oxidizing agent. In particular, understanding whether the steric shielding provided by the long C11–C13 side chains can impart greater stability to oxirane rings compared to less branched analogs represents a key knowledge gap.

Therefore, in this study, we investigated the epoxidation of commercial polyfarnesene using classical and low-cost performic acid epoxidation procedures. The focus was on correlating the epoxidation reaction conditions with the yield of oxirane groups in the polymer and the formation of multifunctional polymers containing more than one type of chemical functional group. By comparing our findings with the behavior of smaller homologues (PB, PI, PM) and plant oil systems reported in the literature, we aim to elucidate the role of the bottlebrush architecture in modulating epoxidation kinetics, oxirane stability, and side reaction pathways. To the best of our knowledge, no prior studies have reported the epoxidation of polyfarnesene using performic acid as an oxidizing agent. This study aims to address this gap.

## 2. Materials and Methods

### 2.1. Materials

Polyfarnesene (liquid farnesene rubber, L-FR-107L) was supplied by Kuraray, Chiyoda City, Japan. Cyclohexane (purity ≥ 99.5%), hydrogen peroxide (purity level ≥ 50%), formic acid (purity ≥ 98%), and sodium carbonate were obtained from Sigma-Aldrich (Saint Louis, MO, USA). All chemicals were used without purification.

### 2.2. Polyfarnesene Epoxidation

Polyfarnesene (60 g) was dissolved in cyclohexane (300 mL) in a 500 mL two-neck round-bottom flask equipped with a reflux condenser. The temperature was set to 60 °C to ensure complete dissolution of the polymer and to provide the thermal energy necessary to drive the epoxidation, given that the rate-determining step of performic acid formation is temperature-dependent [35,36]. The solution was heated to 60 °C under stirring, followed by the addition of formic acid (11.1 mL). An aqueous solution of hydrogen peroxide (50 wt%, 16.7 mL) was then added slowly while maintaining the reaction temperature.

The degree of epoxidation was controlled by varying the reaction time (1, 2, 4, 6, 16, and 20 h), and aliquots (10 mL) were collected at each time interval. The reaction was quenched by adding an aqueous sodium carbonate solution (0.1 M) in portions (approximately 50 mL per washing), typically requiring 2 washes per aliquot, until a neutral pH was reached to neutralize the residual acidity of the reaction mixture. The product was washed with ethanol to induce precipitation and then filtered. The organic phase was concentrated using a rotary evaporator, and the samples were dried in a vacuum oven. For each aliquot, the yield was calculated as the ratio of the recovered polymer mass to the theoretical polymer mass present in the 10 mL aliquot (2 g, based on the initial concentration of 60 g in 300 mL). The yields obtained after neutralization, washing, and drying were 68.2% (1 h), 43.3% (2 h), 50.1% (4 h), 40.4% (6 h), 47.1% (16 h), and 55.3% (20 h).

### 2.3. Characterization of Polymers

#### 2.3.1. Fourier Transform Infrared Spectroscopy

Fourier transform infrared (FTIR) spectroscopy was performed on a Varian Excalibur 3100 FTIR spectrometer using the attenuated total reflectance (ATR) technique in transmission mode in the range of 4000–600 cm^−1^, with a resolution of 2 cm^−1^ and 60 scans.

#### 2.3.2. Nuclear Magnetic Resonance

Epoxidized polyfarnesene samples were examined using nuclear magnetic resonance (^1^H NMR) (Varian Mercury, VX-300, Palo Alto, CA, USA) at 300 MHz. Samples (15 mg) were dissolved in chloroform D1 CDCl_3_ (0.8 mL) in 5 mm NMR tubes at 25 °C.

#### 2.3.3. Gel Permeation Chromatography

The molecular weights of the polymers were determined by means of gel permeation chromatography (GPC) using a Shimadzu chromatograph (Prominence series, Kyoto, Japan) equipped with a refractive index detector and a GPC-803 column (300  mm × 8.0 mm, Shimadzu, Japan). Analyses were performed at 30 °C using chloroform as the eluent at a flow rate of 1.0 mL min^−1^. Sample concentrations of 0.2% m.v^−1^ and injection volumes of 20 μL were used. The number-average molecular weight (M_n_), weight-average molecular weight (M_w_), and polydispersity index (Ð = M_w_/M_n_) were determined using a calibration curve with polystyrene (PS) standards in the range of 700–2.2 × 10^6^.

#### 2.3.4. Differential Scanning Calorimetry

Thermal properties were determined using differential scanning calorimetry (DSC) (Hitachi, High Tech DSC7000 series, Tokyo, Japan). The samples were scanned using DSC with mass samples of approximately 10 mg and a heating temperature range of −100 to 250 °C. All analyses were performed at heating and cooling rates of 10 °C min^−1^ under an inert atmosphere flow (N_2_) of 50 mL min^−1^. This analysis was used to evaluate the glass transition temperatures of the samples.

## 3. Results and Discussion

Epoxidation of polyfarnesene was investigated using performic acid generated in situ from formic acid and hydrogen peroxide. The reaction conditions were chosen based on the kinetic behavior of similar systems and the specific constraints imposed by the polymer architecture. Ramos et al. [35] demonstrated that the epoxidation of 4-methyloct-4-ene, a model compound for 1,4-polyisoprene, follows second-order kinetics, with the formation of performic acid being the rate-determining step. Their study also highlighted the strong influence of temperature and reactant concentration on both the epoxidation rate and the stability of the oxirane rings.

In the present work, a temperature of 60 °C was selected to ensure the complete dissolution of the high-molar-mass polyfarnesene in cyclohexane, which is essential for a homogeneous reaction medium. Moreover, according to Petrović et al. [36], higher temperatures accelerate the epoxidation reaction, allowing for rapid conversion of double bonds. However, their kinetic data also showed that elevated temperatures promote acid-catalyzed ring-opening reactions, leading to secondary products, such as diols and esters [36]. Therefore, the choice of 60 °C represents a compromise: it is sufficiently high to favor fast epoxidation kinetics and polymer solubility; however, the reaction time must be carefully controlled to minimize oxirane degradation and maximize the yield of the epoxidized groups.

Additionally, the reaction mixture was prepared at a relatively high polymer concentration (60 g in 300 mL of cyclohexane) to obtain a substantial amount of functionalized material for subsequent characterization. However, this concentration may introduce diffusional limitations. The high viscosity of the solution and the compact bottlebrush structure of polyfarnesene can hinder the diffusion of hydrogen peroxide and formic acid toward the double bonds, potentially reducing the effective reaction rate and limiting the maximum degree of epoxidation. In contrast to the viscosity-free conditions used in model compound studies, [35], the polymer-based system is likely to be influenced by mass transport phenomena, which must be considered when interpreting the epoxidation yields and the distribution of functional groups along the polymer chains.

### 3.1. Structural Characterization of Polyfarnesene and Its Epoxidized Derivatives

**Figure 1:**
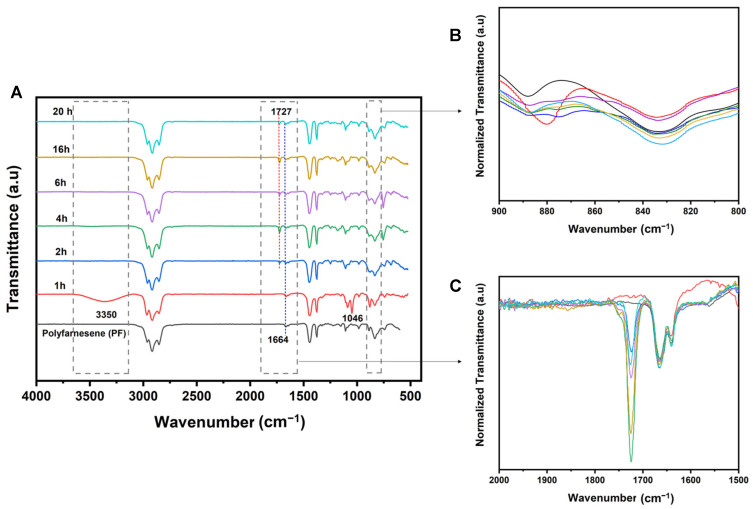
(**A**) FT-IR spectra (offset for clarity) of polyfarnesene and epoxidation products along reaction times. The vertical axis is in arbitrary units (a.u.). Close-up of O-H stretching absorption region; (**B**) close-up of C–O oxirane group deformation region; (**C**) close-up of 2000 cm^−1^–1500 cm^−1^ absorption range region containing bands at 1664 cm^−1^ and 1641 cm^−1^ (isolated double bonds) and 1727 cm^−1^ (ester).

The epoxidation of polyfarnesene was studied using FTIR spectroscopy. Samples were collected at different reaction times to investigate the epoxidation process and monitor the chemical modifications resulting from the reaction. Figure 1A shows the full view of the infrared spectra of polyfarnesene and the epoxidation products. The broad absorption peaks at 2966, 2920, and 2855 cm^−1^ are attributed to the asymmetric stretching vibrations of the −CH_3_, −CH_2_, and −CH groups, respectively. The weak peaks between 1664 and 1641 cm^−1^ are due to the stretching of the structure of the isolated double bond. Absorptions at 1441 and 1376 cm^−1^ are assigned to −CH_2_ and −CH_3_ [2].

The presence of a band at 1664 cm^−1^ in all spectra confirms that a fraction of double bonds remains unreacted even after 20 h. However, the relatively low intensity of this band indicates that the residual unsaturation is moderate compared with the dominant CH and C = O absorptions [33]. Although precise quantification from FTIR alone is not possible because of band broadening and slight shifts caused by changes in the local chemical environment [33]. The persistence of this signal, together with the olefinic resonances observed in the ^1^H NMR spectra, demonstrates that the conversion of C = C bonds is partial rather than exhaustive [37].

In the first hour (red line), the FTIR spectrum presented significant changes. The broad absorption at approximately 3350 cm^−1^ is typically indicative of O–H stretching, indicating the formation of an alcohol group from oxirane ring opening [31,32]. This region showed intense formation of hydroxyl in the first hour (Figure 1A). Another piece of evidence for the presence of hydroxyl groups produced in the first hour of the reaction is the C–O stretching absorption peak at 1046 cm^−1^. This absorption band is highlighted in the spectrum of the first hour of the reaction [32,33].

In the spectrum obtained at the first hour, two specific absorptions related to the oxirane rings were also identified. The first, located at ∼3050 cm^−1^, is attributed to the C–H stretching of the methylene groups of the epoxy ring. It shows low intensity and is very close to the strong O–H absorption. The second absorption band is located at approximately 847–823 cm^−1^, which is attributed to the C–O deformation of the oxirane group (Figure 1B). This absorption band did not increase in intensity with time, suggesting that the oxirane group formed from the reaction with performic acid was quickly ring-opened, generating an ester via reaction with formic acid [32,38].

After 2 h of reaction, an absorption band at 1727 cm^−1^, ascribed to the C = O stretching of the ester, appeared, and the absorption band of the hydroxyl groups disappeared, probably due to the reaction with formic acid. From this reaction time, a progressive increase in the intensity of the ester band was observed with the reaction time (Figure 1C).

### 3.2. Structural Analysis of the Polyfarnesene Epoxidation by Means of ^1^H NMR

**Figure 2:**
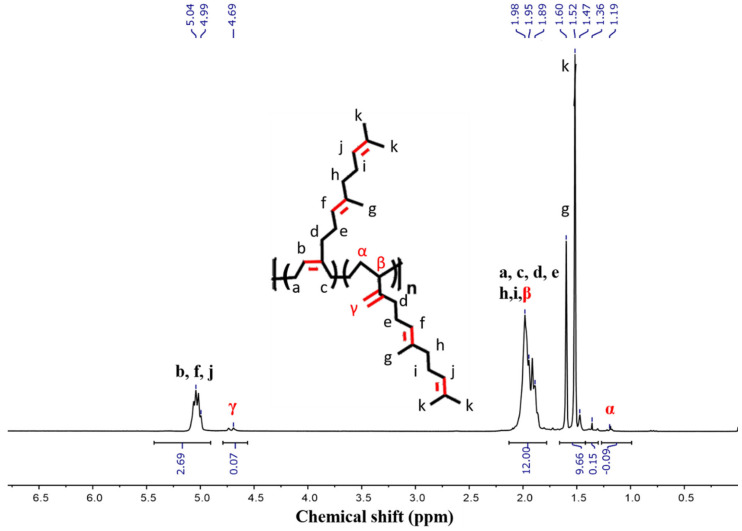
^1^H NMR spectrum of polyfarnesene (300 MHz, CDCl3).

Figure 2 presents the ^1^H NMR spectra of polyfarnesene along with the corresponding assignment of signals to their respective chemical structures. The polyfarnesene has two distinct signals at δ = 5.00–5.25 ppm (b, f, j) and at δ = 4.66–4.93 ppm (γ), revealing the presence of both 1,4- and 3,4-sequences represented in the chemical structure. The signal δ = 5.00–5.25 ppm (b, f, j) is attributed to hydrogens in the double bond, and a signal at δ = 4.66–4.93 ppm (γ) is related to hydrogen in the double bond of configuration 3,4 polyfarnesene. With the integration of signals, the ratio of 1.4/3,4 can be calculated, and it is concluded that 97.3% of polyfarnesene is found in 1,4 configurations. There is no signal for the 1,2 configurations at δ = 5.4–5.8 ppm [39].

In addition, signals were observed at δ = 1.85–2.35 ppm (a, c, d, e, h, i, β) relative to the main and side chains (appearing as broad and multiplet signals) and signals at δ = 1.50–1.75 ppm (g and k) from the methyl groups. Finally, the smaller signal at δ = 1.00–1.25 ppm (α) was attributed to the hydrogens from the methylene group of the 3,4 form [40].

**Figure 3:**
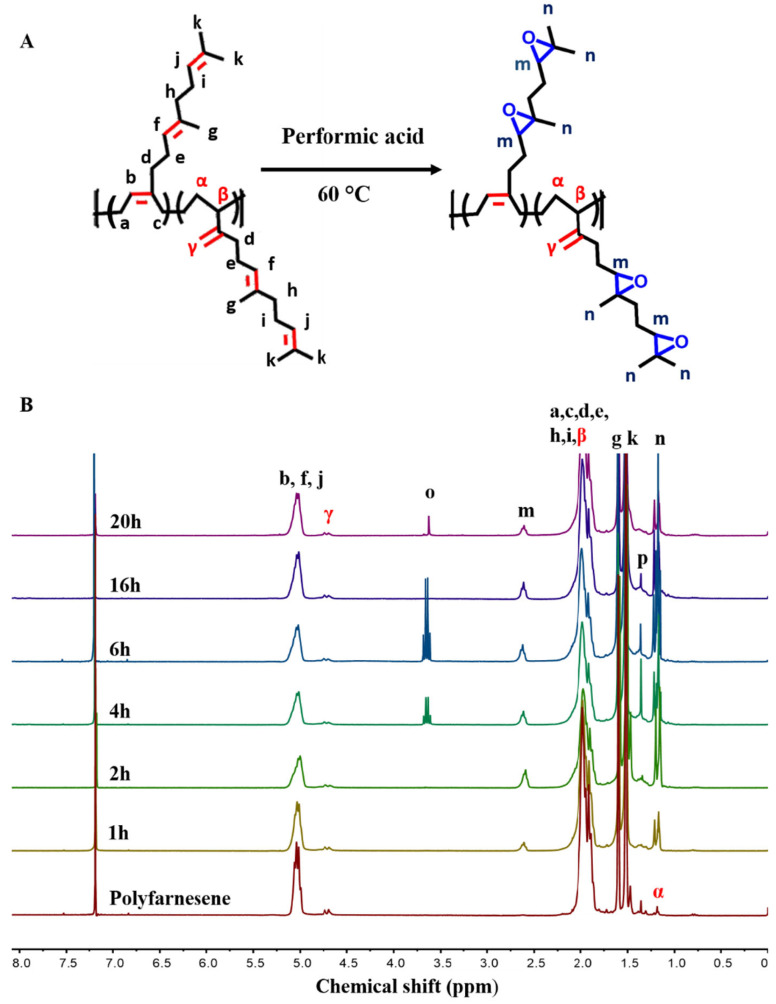
(**A**) Chemical structure of polyfarnesene and epoxidized polyfarnesene. (**B**) ^1^H NMR spectra of polyfarnesene epoxidation products at different reaction times.

Figure 3 shows the ^1^H NMR spectra of the reaction over time. In the initial hours of the reaction, the spectrum shows two additional signals: one at δ = 2.50–2.80 ppm (m), corresponding to hydrogen bonded with carbon in the epoxy ring, and another at δ = 1.00–1.35 ppm (n), attributed to the methyl hydrogens near the oxirane groups. These signals confirm the formation of oxirane rings within the polyfarnesene structure; however, the overlapping of resonances from different isoprene units prevents an unambiguous assignment of the oxirane location exclusively to the side chains.

The signal corresponding to the oxirane rings, which are substituted at δ = 2.50–2.80 ppm (m), increased in the first 6 h and then decreased, indicating a ring-opening reaction. The methyl hydrogens bonded to the oxirane ring give a signal at δ = 1.00–1.35 ppm (n). Although a signal at δ = 3.50–3.80 ppm (o) was initially attributed to a potential ethanol contaminant, the integration values did not significantly support the hypothesis that this contaminant contributed to the methyl signal at δ = 1.00–1.35 ppm (n). Therefore, the spectral changes primarily reflect the progressive chemical transformation of oxirane functionality, rather than significant interference from impurities.

^1^H NMR spectra revealed additional resonances consistent with side reactions of the oxirane groups during the epoxidation of polyfarnesene. After 16 h, the disappearance of the multiplet signal at δ = 3.50–3.80 ppm (o) can be explained by the secondary esterification of the hydroxyl groups with formic acid present in the medium, yielding ester groups within the polymer structure. This interpretation is supported by the emergence of a signal at δ = 1.25–1.50 ppm (p), which is consistent with the methyl groups of α-branched esters, as well as by the characteristic proton of formic esters expected at approximately δ = 8.00 ppm [41]. This signal can be identified by expanding this region (Supplementary Materials Figure S1), in which it is evident that the opening reaction starts earlier (4 h) than expected. Unfortunately, owing to the low intensity of this signal, the quantitative evaluation of the degree of ester formation was not possible. Nevertheless, infrared spectral analysis supports the hypothesis of ester formation.

### 3.3. Quantitative Analysis of Polyfarnesene Epoxidation by Means of ^1^H NMR

The presence of the main double bond signals at δ = 5.00–5.25 ppm (b, f, j) suggests that a considerable quantity of these groups remained unreacted [42,43]. Notably, even the signal corresponding to the 3,4 microstructure (γ) remains visible with no significant alterations throughout the reaction. This observation indicates that epoxidation occurred preferentially at specific sites within the polymer structure. The persistence of these signals suggests that the main-chain double bonds may be less accessible to the epoxidizing agent, possibly due to steric hindrance or conformational constraints within the polymer backbone [30,31]. Furthermore, similar chemical shifts and signal overlap between different isoprene units prevent a definitive assignment of whether the newly formed oxirane rings are located exclusively in the side chains or also involve some main-chain units that underwent chemical shift changes upon epoxidation [43,44,45].

Terminal alkenes have reduced steric hindrance at the double bond, facilitating the approach of reagents and enabling faster reaction rates. In contrast, internal alkenes exhibit enhanced thermodynamic stability due to hyperconjugation, in which electrons from adjacent sp^3^-hybridized C–H or C–C σ-bonds delocalize into the π* orbital of the double bond [44,46]. This stabilization is significantly less pronounced in terminal alkenes, which contain fewer adjacent substituents capable of participating in hyperconjugative interactions. Consequently, terminal alkenes display higher heats of hydrogenation and exhibit increased susceptibility to electrophilic attack under certain conditions, such as epoxidation of polydienes, in which the kinetic selectivity for less congested terminal sites often overrides the greater electronic nucleophilicity of internal alkenes, as observed for bulky oxidants and catalysts [26,30,47,48]. In summary, these structural characteristics account for the enhanced reactivity of monosubstituted alkenes relative to their internal counterparts [44].

The specific signal expected for hydrogens from the oxirane ring formation in monosubstituted alkenes (3,4 microstructure) typically appears between δ = 2.85 and 3.00 ppm [28]. In the present study, no significant signal was observed in this region, suggesting that these double bonds remained largely unreacted under the conditions employed. This observation is consistent with the report by Matic et al. [16] for the epoxidation of polymyrcene, wherein similar considerations regarding the unreacted double bonds of the 3,4 microstructure (γ) were discussed.

To quantify the extent of epoxidation, two complementary approaches based on ^1^H NMR integration were employed, following a method adapted from Matic et al. [16] (Equation (1)). This equation considers the hydrogen atoms in the epoxide ring (signal “m”) as the product and the sum of hydrogen atoms bonded to double bonds in both the main and side chains (signals “b,” “f,” “j, “ and “γ”) as the reactants. The signal “γ” is divided by two to account for the two hydrogen atoms on the same carbon, where the signal intensity is doubled but corresponds to a single reacting unit.

The equation of Matic et al. [16] (Equation (1)) was used to determine the degree of epoxidation in polymyrcene and evaluate the degree of epoxidation of polyfarnesene.

(1) $$\mathrm{Degree}\;\mathrm{of}\;\mathrm{epoxidation}=\frac{Int(m)}{\left[Int\left(b,f,j\right)+\frac{Int\left(\gamma \right)}{2}+Int\left(m\right)\right]}$$

Equation (1) provides an estimate of the overall degree of epoxidation across all double bonds in the polymer structure.

Additionally, a second quantification method was developed using only the methyl group signals (Equation (2)). In this approach, signals “g” and “k” (δ = 1.50–1.75 ppm) correspond to methyl groups originally attached to C = C double bonds, whereas signal “n” (δ = 1.00–1.25 ppm) represents methyl groups in the vicinity of the newly formed oxirane rings. The ratio calculated using Equation (2) reflects the fraction of double bonds converted into epoxy groups, as expressed below:

(2) $$\mathrm{Degree}\;\mathrm{of}\;\mathrm{epoxidation}\;=\;\frac{Int(n)}{\left[Int\left(n\right)+Int\left(g+k\right)\right]}$$

Notably, Equation (2) is particularly sensitive to structural changes near the methyl-bearing carbons, which are predominantly located in the side chain units. However, owing to signal overlap and the complexity of the polyfarnesene microstructure, this approach cannot unambiguously distinguish between the oxirane rings formed in the side chains and those in the main-chain units that underwent chemical shift changes upon epoxidation. Therefore, Equation (2) should be interpreted as an estimate of epoxidation occurring in environments associated with the methyl groups, rather than as a definitive measure of the side-chain-specific reaction yield.

**Figure 4:**
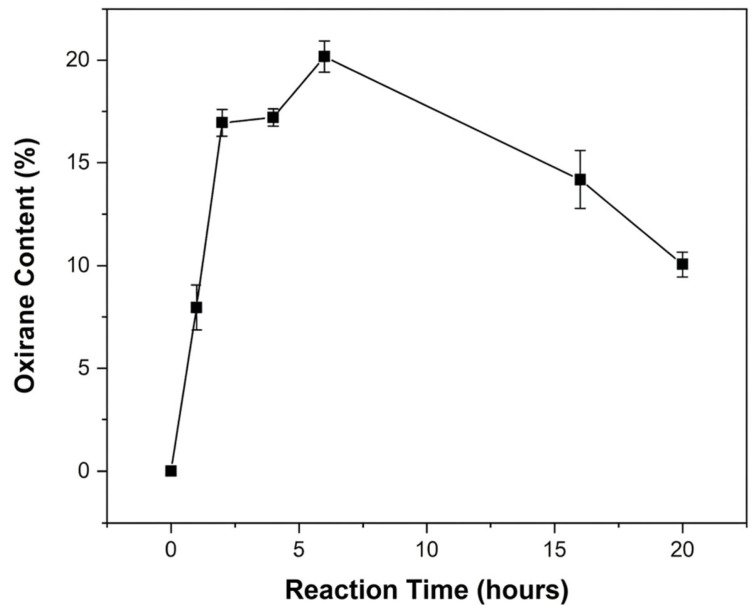
Degree of epoxidation of polyfarnesene over time.

A comparison between the results obtained from Equations (1) and (2) provides insight into the distribution of the epoxidized units across different microstructural environments. The quantification of oxirane groups using both calculation methods and NMR processing software MestreNova (version 12.0.0, Mestrelab Research, Santiago de Compostela, Spain) is summarized in Figure 4, which presents the values and standard deviations of the oxirane yield. The complete dataset is provided in Supplementary Materials Table S1.

Each data point in Table S1 represents the mean oxirane content obtained from three repeated integrations, and the associated standard deviations were used to estimate the standard deviation of each method before performing the statistical comparison.

To quantitatively assess the agreement between the two NMR-based quantification approaches, the oxirane contents obtained from Equations (1) and (2) at each reaction time (Table S1) were compared using a paired two-tailed Student’s *t*-test (OriginPro, OriginLab, Northampton, MA, USA). The test yielded *t(6)* = −1.74 and *p* = 0.13, indicating no statistically significant difference between the two calculation methods at the 95% confidence level. Therefore, within the experimental standard deviation, both Equations (1) and (2) provide mutually consistent oxirane contents, and the observed discrepancies, particularly at 2 h, are interpreted as a consequence of their different sensitivities to distinct structural environments within the polymer, in a regime where epoxidation and ring-opening side reactions occur simultaneously.

The degree of epoxidation increased rapidly, reaching ~20% at approximately 6 h, and subsequently decreased progressively to ~14% at 16 h and ~10% at 20 h. Although partial degradation of the oxirane rings was evident, the overall rate of their consumption was slower than that typically reported for similar systems undergoing acid- or base-catalyzed ring opening. [30,47] Therefore, the decline after 6 h was attributed to competitive side reactions of the oxirane groups with formic acid, in agreement with the progressive increase of signals “n” and “m,” followed by their partial decrease and the concomitant appearance of signal “p” in the ^1^H NMR spectra.

### 3.4. Epoxidation Mechanism and Oxirane Ring Stability

The epoxidation of alkenes by peracids proceeds via the classical Prilezhaev mechanism [49]. In our system, performic acid is generated in situ from formic acid and hydrogen peroxide (HCO_2_H + H_2_O_2_ ⇌ HCO_3_H + H_2_O). The equilibrium between the formation of performic acid and the reverse reactions governs its concentration; thus, it does not directly correspond to the stoichiometric amounts of H_2_O_2_ and HCO_2_H added but depends on the H_2_O_2_/acid ratio, acidity, temperature, and reaction time [50]. The complexity of the system is further amplified by the high viscosity of the polymer matrix and its bottlebrush architecture, which impose mass transfer limitations.

Formic acid is essential for promoting epoxidation. In the absence of carboxylic acid (such as formic or acetic acid) [50], H_2_O_2_ alone does not generate epoxides under these conditions. Therefore, a “no formic acid” control experiment would not produce epoxides, rendering a direct comparison uninformative regarding the origin of the oxirane ring-opening.

Similar results were obtained by Habri [30], who investigated the autocatalytic degradation of the oxirane ring by performic acid during the epoxidation of oleic acid. They found that a higher formic acid content could lead to an unstable oxirane ring. At a formic acid–oxirane molar ratio of 2:1, the oxirane content rapidly decreased due to acid cleavage and ring opening for up to 60 min. At other molar ratios, low concentrations of formic acid can increase and decrease the oxirane ring percentage over the reaction time.

In this study, the instability of the reaction was also observed, and even considering the relatively high standard deviation, it is clear that the signal relative to the oxirane rings decreased after 6 h of reaction.

Some studies [11,31,32] have reported rapid oxirane ring opening, which typically occurs within minutes or a few hours; in this work, the degradation rate observed was considerably slower. Even after 20 h of reaction, approximately 10% of the oxirane NMR signal remained, which corresponded to nearly 50% of the maximum yield obtained at 6 h. Therefore, our results indicated that polyfarnesene oxirane rings formed by performic acid participate in side reactions, generating other functional groups in the polymer chain. They exhibited greater stability than similar systems reported in the literature [33].

**Figure 5:**
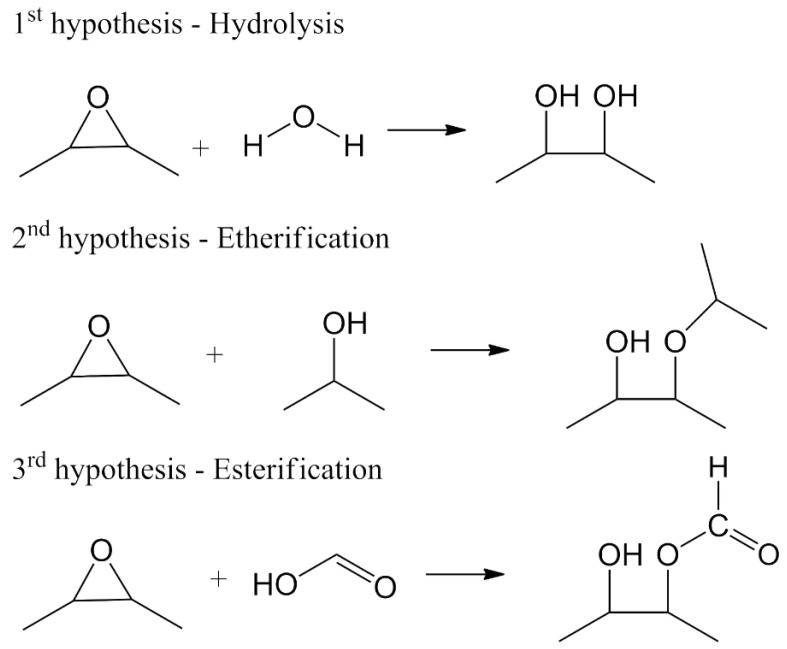
Proposed main reaction pathways responsible for the consumption of epoxy groups: (1) hydrolysis leading to vicinal diols; (2) etherification with alcohols; and (3) esterification with carboxylic acids.

Figure 5 illustrates the different reaction pathways that can consume oxirane groups. The first hypothesis (hydrolysis) states that water molecules present in the heated system open the epoxy ring, generating vicinal hydroxyl groups. The second hypothesis (etherification) results from the reaction between epoxy groups and free hydroxyls (originating from hydrolysis or pre-existing in the formulation), forming ether bonds and new β-hydroxyl groups, and eventually crosslinking. The third hypothesis (esterification) states that epoxy groups react directly with carboxylic acid groups, leading to the formation of β-hydroxy esters. Under elevated temperature conditions, residual -COOH can also react with available hydroxyls [11].

Combining the NMR and FTIR spectra results and according to the hypotheses of the epoxidation reactions studied, it can be concluded that epoxidation occurs in the first hour, but at the same time, the opening of rings starts creating hydroxyl groups, which have a short lifetime because they are converted into ester groups with formic acid. The ester was the final product of the reaction when the concentration increased with time.

Oxirane rings are promptly formed; however, they gradually open and undergo esterification. Some double bonds resisted for hours, indicating protection for this group in the polymer structure. Following the results of ^1^H NMR, the content of oxirane rings reached approximately 20% after 6 h, decreasing to 10% after 20 h. Even after hours, there were many double bonds in the polymer, indicating that some of the double bonds were not accessible to the performic acid.

This enhanced stability can be rationalized by considering the homologous series of polydienes and terpenes, including polybutadiene (PB), polyisoprene (PI), polymyrcene (PM), and polyfarnesene (PF). The primary structural distinction among these polymers lies in the length of their side chains: PB has no side chains, PI bears a methyl group (C1), PM possesses C6–C8 side chains, and PF is characterized by C11–C13 branches, conferring a unique bottlebrush architecture [12,17].

Studies on analogous systems confirm distinct reaction dynamics and structural stability. For instance, Zhou et al. [51] reported that the epoxidation of PB with mCPBA at 30 °C was complete within 1 h, and subsequent oxirane ring cleavage using periodic acid occurred rapidly, between 0.5 and 2 h. Matic and Schlaad [23] observed that while the epoxidation of polymyrcene is nearly quantitative, the hydrolysis of oxirane rings to form diols is often incomplete. This suggests that the increased side chain length in PM (C6) provides some steric protection to the oxirane rings compared to the methyl group in PI. In plant oil-based systems, which have less sterically hindered structures, oxirane ring opening by acids typically occurs within minutes or a few hours [33].

In contrast, polyfarnesene retains approximately 10% of the oxirane signal even after 20 h of reaction. This slower degradation kinetics is attributed to the massive steric hindrance imposed by the bottlebrush architecture of PF. The long C11–C13 side chains act as a “shield,” hindering the approach of nucleophilic reagents (such as formic acid or water) to the oxirane rings located within the polymer structure. Therefore, the unique molecular architecture of polyfarnesene directly influences its reactivity, making it distinct from smaller homologues and contributing to the prolonged stability of oxirane groups observed in this study.

### 3.5. Effect of Epoxidation on the Molecular Weight Distribution of Polyfarnesene by Means of Gel Permeation Chromatography

**Figure 6:**
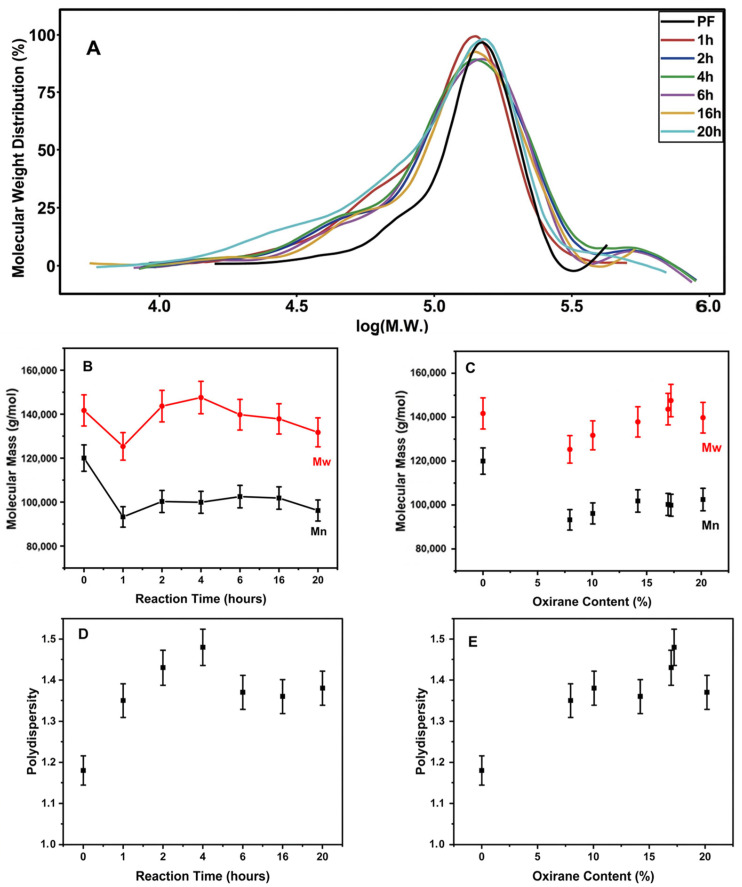
(**A**) Molecular weight distribution of polyfarnesene (PF) and epoxidized polyfarnesene as a function of oxirane content (%). (**B**) Weight- and number-average molecular weight versus reaction time; (**C**) weight- and number-average molecular weight versus oxirane content; (**D**) polydispersity of sample versus reaction time; (**E**) polydispersity versus oxirane content.

Gel permeation chromatography allows the determination of the molar mass of polyfarnesene and the evaluation of changes due to the epoxidation reaction. Figure 6 shows the number-average molar weight (M_n_), weight-average molar weight (M_w_), polydispersity (D), and molecular weight distribution of polyfarnesene as a function of the epoxidation content. The quantitative values are provided in the Supplementary Materials.

As shown in Figure 6A, a slight shift in the peaks of epoxidized polyfarnesene compared to those of polyfarnesene is observed, indicating a variation in the molecular weight. Changes in the epoxidation content did not significantly influence the profiles of the curves, showing similar behavior for all contents. Considering the curve profile, no indication of crosslinks between the polymer chains due to intramolecular reactions was noted. This type of reaction can be exemplified by the formation of ether bonds (Figure 5, second hypothesis). Similarly, no significant main-chain fragmentation was detected.

Considering only the reaction time (Figure 6B), after epoxidation, the molecular weight slightly decreased in the first hour, probably because of a reduction in the hydrodynamic volume of the polyfarnesene molecular chains. M_n_ and M_w_ slightly increased over time; however, the difference in molecular weight was small. Polydispersity increased (Figure 6D), showing that the epoxidation reaction affected the homogeneity of the molecular chain distribution, suggesting structural changes but with a low impact of the reaction time after the first hour.

When M_w_ was plotted as a function of the oxirane content (Figure 6C), a decrease in M_w_ and M_n_ was observed in the first hour, but a linear increase with oxirane content was observed over time. Figure 6C shows that after the initial contraction of the epoxidized polymer (reduction in the hydrodynamic radius), the molar weight of the epoxidation sample increased with the oxirane content. Although the M_w_ value at approximately 20% oxirane content shows a slight deviation from the linear trend, this variation remains within the experimental error of the GPC measurements. Similarly, the slight plateau observed in M_n_ between 15% and 20% oxirane content is modest and consistent with the narrow variation range observed across the epoxidation series.

Considering the polyfarnesene structure, each repeating unit contains three double bonds distributed across different microstructural environments, including the main-side-chain units [12,17]. Based on the molar mass of β-farnesene (204 g·mol^−1^) and the weight-average molar mass (M_w_) of polyfarnesene (142,000 g·mol^−1^), the total number of double bonds per polymer chain was estimated.

Each polymer molecule comprises approximately 695 farnesene repeating units, corresponding to a total of ~2085 double bonds. Using the degree of epoxidation determined by means of ^1^H NMR (~20% maximum yield), the number of oxirane rings formed per chain was calculated to be approximately 417. This value represents the total epoxidized units, regardless of their specific location within the polymer microstructure.

The observed maximum epoxidation yield of ~20% indicates that approximately 417 out of 2085 double bonds per chain were converted into oxirane rings. Given the distribution of double bonds across different microstructural environments in polyfarnesene, it is plausible that epoxidation occurs preferentially in more accessible regions of the polymer [12,17]. However, the available spectroscopic data do not allow definitive assignment of whether these oxirane rings are located predominantly in side chains, main-chain units, or distributed across both environments.

The appearance of hydroxyl and ester signals in the ^1^H NMR spectra indicates that the oxirane ring-opening and esterification reactions occur concurrently with epoxidation. These side reactions are likely favored at less sterically hindered positions, which may include oxirane rings formed in more accessible regions of the polymer structure. The steric constraints imposed by the polymer backbone may influence the relative rates of epoxidation versus ring opening at different sites, potentially explaining why hydroxylated and esterified species form rapidly, even when a substantial fraction of double bonds remains unreacted.

### 3.6. Impact of Oxirane Content on Glass Transition Temperature (T_g_)

Glass transition is one of the most important properties of polymers, which is associated with the mobility of the polymer chain. Supplementary Materials Table S2 presents the differential scanning calorimetry (DSC) data, showing the glass transition temperatures for each product of the reaction.

**Figure 7:**
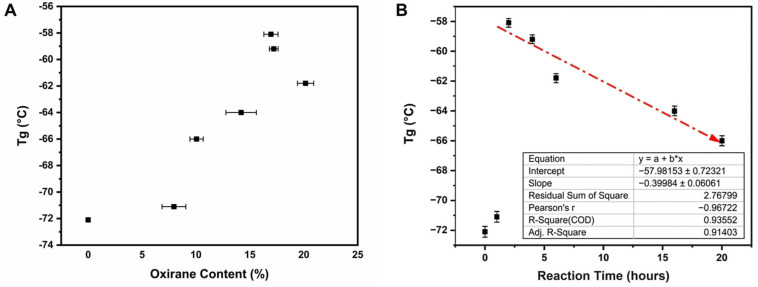
(**A**) Glass transition temperature (T_g_) as a function of oxirane content, highlighting the progressive increase of in T_g_ with higher epoxidation degree. (**B**) Evolution of T_g_ with reaction time, showing the abrupt increase at 2 h followed by a quasi-linear decrease due to competitive side reactions and formation of hydroxyl and ester groups from performic acid. The red dash-dotted line represents the fit to the equation y=a+bx, with parameters provided in the legend.

At first glance, T_g_ increases as the oxirane content increases (Figure 7A). This result is similar to that obtained by Matic et al. for the T_g_ of epoxidized polymyrcene [16,52] and that observed by Dias et al. for the epoxidation of syndiotactic polybutadiene [53]. This result shows that the introduction of oxirane rings into the polymer structure increases T_g_, probably because of the restricted chain mobility imposed by the steric hindrance and polar interactions of the epoxy groups, as the introduction of these polar segments significantly modified T_g_ and chain mobility [54,55]. Likewise, in vitrimer-like polyhydroxyesters, Cunha et al. [56] observed that the progressive formation of hydroxyl and ester groups derived from epoxy ring-opening reactions directly influenced the glass transition and thermal stability of the network.

Nevertheless, an interesting result appears when T_g_ is plotted as a function of reaction time. T_g_ abruptly increases at two hours and begins to decrease quasi-linearly as the reaction time increases (Figure 7B). As revealed by the FTIR and NMR analyses, after a few hours, the oxirane rings form hydroxyl and ester groups. The incorporation of these polar functional groups reduces the structural contribution of the oxirane rings, alters chain packing, and increases segmental mobility, which explains the progressive decrease in the glass transition temperature of the multifunctional polymer as a function of reaction time.

## 4. Conclusions

In this study, commercial polyfarnesene was epoxidized using in situ-generated performic acid with hydrogen peroxide. The formation of oxirane rings reached a maximum content of approximately 20% within 6 h, but this concentration tended to decrease owing to the onset of side reactions in the first few hours, resulting in the formation of hydroxyl and formic acid ester groups. The observed limitation of approximately 20% epoxidation yield suggests that a significant fraction of double bonds remains unreacted under the conditions employed. This may be attributed to the complex bottlebrush architecture of polyfarnesene, wherein steric constraints imposed by the polymer structure could limit the accessibility of certain double bonds to the epoxidizing agent. Additionally, spectroscopic evidence indicates that once oxirane rings are formed, they are susceptible to ring-opening and esterification side reactions, which consume the epoxy groups and contribute to the plateau in yield. The bottlebrush architecture of polyfarnesene appears to influence the reaction kinetics, potentially limiting the maximum epoxidation efficiency and favoring competitive side reactions over further epoxy formation. However, definitive conclusions about the precise location of oxirane rings (whether in the side chains, main chain, or distributed across different microstructural units) cannot be drawn from the available data owing to signal overlap in ^1^H NMR spectra and the limitations of techniques such as GPC for providing spatially resolved information. Regarding the impact on properties, epoxidation increases the glass transition temperature (T_g_) of the resulting polymers, as observed for other dienes, such as polymyrcene. However, as side reactions proceed, the formation of ester and hydroxyl groups tends to moderate the increase in T_g_, and in practice, T_g_ reaches a maximum before declining at longer reaction times. Therefore, if products with higher T_g_ are desired, side reactions should be minimized. For this purpose, reactions could be carried out under milder conditions to reduce the rate of side reactions or even conducted in solvents to maximize the expansion of the polyfarnesene molecular and bottlebrush structures and improve the reaction of the innermost backbone double bonds, thereby lowering the tendency for side reactions. In summary, this study confirms that polyfarnesene undergoes the same fundamental epoxidation and side reactions as other polydienes. However, the key finding is that its bottlebrush architecture dramatically slows oxirane degradation through steric shielding, representing a novel contribution to understanding structure–reactivity relationships in branched polymers.

## Data Availability

The original contributions presented in this study are included in the article/Supplementary Materials. Further inquiries can be directed to the corresponding author.

## Supplementary Materials

The following supporting information can be downloaded at https://www.mdpi.com/article/10.3390/polym18070844/s1. Figure S1: ^1^H NMR spectra, Close-up of the region of signal of formic hydrogen. The signal starting with 2 h; Table S1: Quantification of oxirane content determined by ^1^H NMR; Table S2: Tg of polyfarnesene epoxidized at different reaction times.

## Abbreviations

The following abbreviations are used in this manuscript:

FTIR	Fourier transform infrared spectroscopy
NMR	Nuclear Magnetic Resonance
GPC	Gel Permeation Chromatography
M_n_	Number-average molecular weight
M_w_	Weight-average molecular weight
Ð	Polydispersity index
DSC	Differential Scanning Calorimetry
T_g_	Glass transition
PF	Polyfarnesene
PB	Polybutadiene
PI	Polyisoprene
PM	Polymyrcene
