# Supplementary material for: In Situ Performic Acid Epoxidation of Polyfarnesene: Evidence of Oxirane Ring Instability and Its Impact on Multifunctional Polymer Composition
Source: Polymers (Basel). 2026 Mar 30;18(7):844. doi: 10.3390/polym18070844 (PMC13074262; doi:10.3390/polym18070844)
Supplement: Supplementary file 1 [file polymers-18-00844-s001.zip › polymers-4211113-supplementary.pdf]

polymers

Supporting Information

In Situ Performic Acid Epoxidation of Polyfarnesene: Evidence of Oxirane Ring Instability and Its Impact on Multifunctional Polymer Composition

Geilza A. Porto [1], Luiz Guilherme A. de Paula [1], Luciano N. Batista [2] and Marcos L. Dias [1,*]

[1] Instituto de Macromoléculas Professora Eloisa Mano (IMA), Universidade Federal do Rio de Janeiro, Av. Horacio Macedo, Bloco J, 2030, Rio de Janeiro 21941-598, RJ, Brazil; geilzaporto@ima.ufrj.br (G.A.P.); luizguilhermeacm@ima.ufrj.br (L.G.A.d.P.)
[2] Instituto Nacional de Metrologia, Qualidade e Tecnologia (Inmetro), Avenida Nossa Senhora das Graças, 50, Duque de Caxias 25250-020, RJ, Brazil; lnbatista@inmetro.gov.br
* Correspondence: mldias@ima.ufrj.br

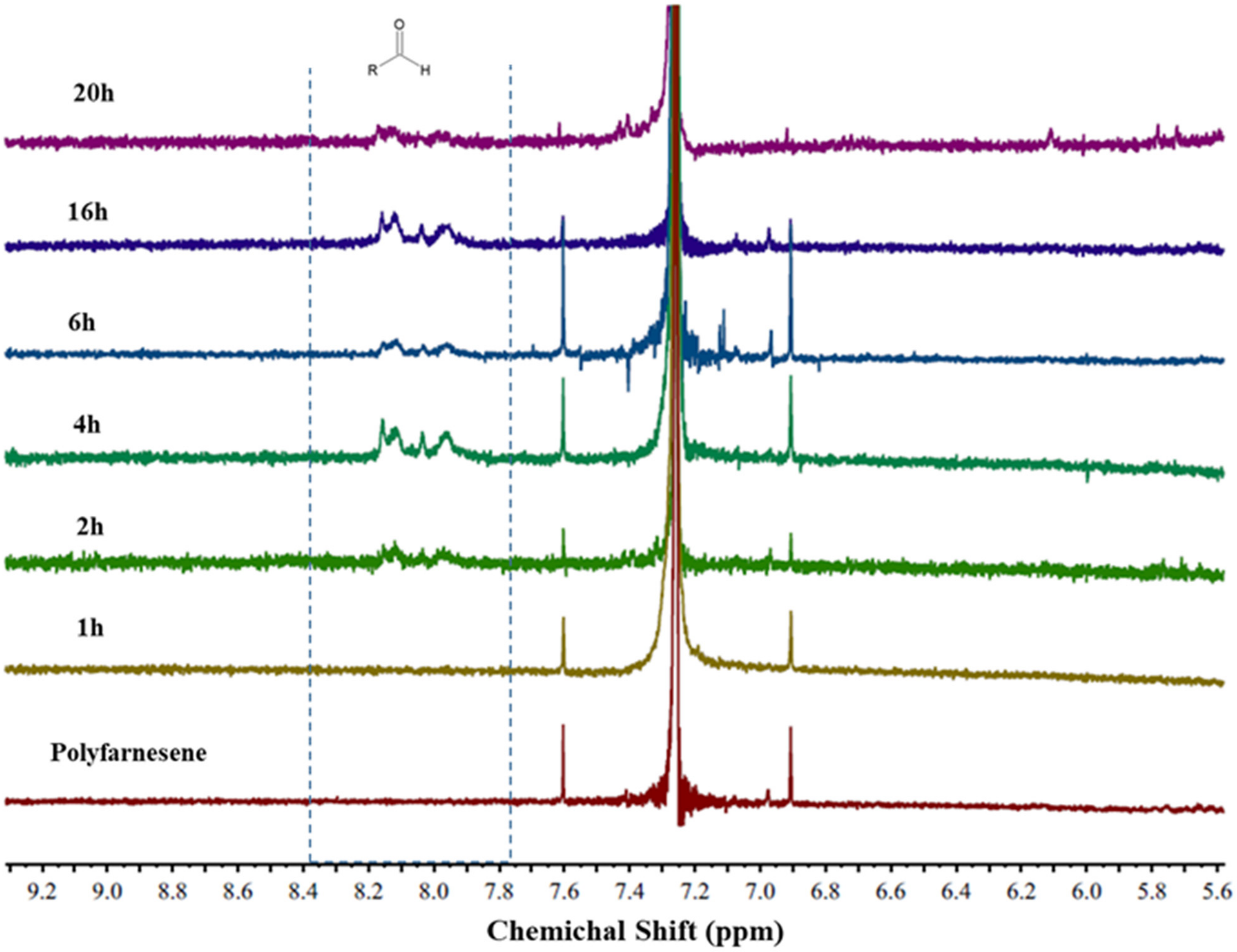

Figure S1. ^{1}H NMR spectra, close-up of the region of signal of formic hydrogen. The signal starting with 2 hours.

Table S1. Quantification of oxirane content determined by ^{1}H NMR.

Time (h)	Oxirane (%) Equation 1	SD Equation 1	Oxirane (%) Equation 2	SD Equation 2
**0**	0.00	0.00	0.00	0.00
**1**	8.87	2.93	7.96	1.10
**2**	23.49	1.73	16.96	0.66
**4**	19.85	3.35	17.22	0.40
**6**	20.49	0.03	20.16	0.76
**16**	14.21	0.44	14.19	1.41
**20**	11.90	7.21	10.06	0.61

SD = standard deviation from three repeated integrations

Table S2. T_g of polyfarnesene epoxidized at different reaction times.

Time (h)	T_g (°C)	SD
**0**	-72.00	0.36
**1**	-71.00	0.35
**2**	-58.00	0.29
**4**	-59.00	0.30
**6**	-62.00	0.31
**16**	-64.00	0.32
**20**	-67.00	0.33

SD = standard deviation from three repeated integrations
